# Development and Validation of a Novel Acute Myeloid Leukemia–Composite Model to Estimate Risks of Mortality

**DOI:** 10.1001/jamaoncol.2017.2714

**Published:** 2017-09-07

**Authors:** Mohamed L. Sorror, Barry E. Storer, Amir T. Fathi, Aaron T. Gerds, Bruno C. Medeiros, Paul Shami, Andrew M. Brunner, Mikkael A. Sekeres, Sudipto Mukherjee, Esteban Peña, Mahmoud Elsawy, Shylo Wardyn, Jennifer Whitten, Rachelle Moore, Pamela S. Becker, Jeannine S. McCune, Frederick R. Appelbaum, Elihu H. Estey

**Affiliations:** 1 Clinical Research Division, Fred Hutchinson Cancer Research Center, University of Washington School of Medicine, Seattle; 2 Division of Medical Oncology, Department of Medicine, University of Washington School of Medicine, Seattle; 3 Clinical Statistics Program, Clinical Research Division, Fred Hutchinson Cancer Research Center, University of Washington School of Public Health, Seattle; 4 Department of Biostatistics, University of Washington School of Public Health, Seattle; 5 Massachusetts General Hospital, Harvard Medical School, Boston; 6 Leukemia & Myeloid Disorders Program, Cleveland Clinic, Cleveland, Ohio; 7 Department of Medicine, Division of Hematology, Stanford University, Stanford, California; 8 Huntsman Cancer Institute, Division of Hematology and Hematologic Malignancies, University of Utah, Salt Lake City; 9 Medical Oncology, National Cancer Institute, Cairo University, Cairo, Egypt

## Abstract

**Importance:**

To our knowledge, this multicenter analysis is the first to test and validate (1) the prognostic impact of comorbidities on 1-year mortality after initial therapy of acute myeloid leukemia (AML) and (2) a novel, risk-stratifying composite model incorporating comorbidities, age, and cytogenetic and molecular risks.

**Objective:**

To accurately estimate risks of mortality by developing and validating a composite model that combines the most significant patient-specific and AML-specific features.

**Design, Setting, and Participants:**

This is a retrospective cohort study. A series of comorbidities, including those already incorporated into the hematopoietic cell transplantation–comorbidity index (HCT-CI), were evaluated. Patients were randomly divided into a training set (n = 733) and a validation set (n = 367). In the training set, covariates associated with 1-year overall mortality at a significance level of *P* < .10 constructed a multivariate Cox proportional hazards model in which the impact of each covariate was adjusted for that of all others. Then, the adjusted hazard ratios were used as weights. Performances of models were compared using C statistics for continuous outcomes and area under the curve (AUC) for binary outcomes.

**Exposures:**

Initial therapy for AML.

**Main Outcomes and Measures:**

Death within 1 year after initial therapy for AML.

**Results:**

A total of 1100 patients, ages 20 to 89 years, were treated for AML between January 1, 2008, and December 31, 2012, at 5 academic institutions specialized in treating AML; 605 (55%) were male, and 495 (45%) were female. In the validation set, the original HCT-CI had better C statistic and AUC estimates compared with the AML comorbidity index for prediction of 1-year mortality. Augmenting the original HCT-CI with 3 independently significant comorbidities, hypoalbuminemia, thrombocytopenia, and high lactate dehydrogenase level, yielded a better C statistic of 0.66 and AUC of 0.69 for 1-year mortality. A composite model comprising augmented HCT-CI, age, and cytogenetic/molecular risks had even better predictive estimates of 0.72 and 0.76, respectively.

**Conclusions and Relevance:**

In this cohort study, comorbidities influenced 1-year survival of patients with AML, and comorbidities are best captured by an augmented HCT-CI. The augmented HCT-CI, age, and cytogenetic/molecular risks could be combined into an AML composite model that could guide treatment decision-making and trial design in AML. Studying physical, cognitive, and social health might further clarify the prognostic role of aging. Targeting comorbidities with interventions alongside specific AML therapy might improve survival.

## Key Points

**Question:**

Can a model incorporating patient-specific (comorbidities and age) and acute myeloid leukemia (AML)-specific features (cytogenetic and molecular alterations) predict mortality after AML treatment?

**Findings:**

In a multicenter cohort study of 1100 patients, we demonstrated that (1) comorbidities had a significant impact on 1-year mortality after initial therapy for AML, (2) an augmented hematopoietic cell transplant–comorbidity index (HCT-CI) was the best suited index for comorbidity evaluation in AML, and (3) an AML composite model of augmented HCT-CI, age, and cytogenetic/molecular risks has a strong AUC of 0.76 for 1-year mortality.

**Meaning:**

An AML composite model can guide decision-making about treatment of AML.

## Teaser

This cohort study examines the prognostic association of comorbidities on 1-year mortality after initial therapy of acute myeloid leukemia and a risk-stratifying composite model that incorporates comorbidities, age, and cytogenetic/molecular risks.

## Introduction

Acute myeloid leukemia (AML) is the most common form of acute leukemia,^1^ and the number of new cases are rising annually.^2^ The 5-year survival rate is only 26.6%.^2^ Survival rates are even lower among patients ages 65 to 74 years (5.3%) and 75 years or older (1.6%), who together constitute more than 50% of new AML cases and who tend to have a greater burden of comorbidities^3^ and more profound limitations in physical health and robustness^4^ than younger patients.

Mortality in AML, in part, reflects its inherent resistance to therapy and, in part, the deleterious, and at times, lethal effects of treatment. Quantifying both of these risks is critical to the fundamental decision of whether patients should receive any specific therapy and, if so, whether it should be less or more “intense.” Therapy guidelines, such as those of the National Comprehensive Cancer Network (NCCN), have tended to focus largely on an arbitrary age cutoff of 60 or 65 years as the arbiter of the appropriateness of therapeutic intensity.^6^ While performance status might be used to refine decision making,^7^ it does not differentiate between functional impairment due to AML, which is potentially responsive to anti-AML treatment, and that due to comorbidities, which may pose possible contraindications to intensive treatment.^8^

We have shown that accounting for comorbidities can provide valuable information on the risks associated with allogeneic hematopoietic cell transplantation (HCT).^9^ However, the prognostic importance of comorbidities existing at the time of AML diagnosis has not been systematically examined regarding their impact for choosing initial treatment for AML. Indeed, patients with comorbidities are often simply excluded from clinical trials precluding a careful evaluation of their relevance. Herein, we sought to determine whether (1) comorbidities have an impact on 1-year mortality in patients newly diagnosed as having AML, (2) a new AML-specific comorbidity index (CI) can be designed and validated to outperform the HCT-CI in patients presenting for initial therapy, and (3) a model incorporating comorbidities together with age and cytogenetic and/or molecular risks could be designed and validated to improve prognostic evaluation for such patients with AML.

## Methods

We followed the Enhancing the QUAlity and Transparency of Health Research (EQUATOR) reporting guidelines using the Transparent Reporting of a Multivariable Prediction Model for Individual Prognosis Or Diagnosis (TRIPOD) criteria.^12^

### Source of Data

This is a retrospective hospital-based cohort study of data collected by review of electronic medical records and computer databases for 1100 patients. All patients were consecutively and concurrently treated at each of the AML specialty centers between January 1, 2008, and December 31, 2012. Data were randomly divided into a training set (n = 733) and a validation set (n = 367) for development and validation of the model, respectively. This protocol was approved by the institutional review boards of the Fred Hutchinson Cancer Research Center (FHCRC) and the collaborating sites. All data regarding treatment and demographic information were utilized according to the Declaration of Helsinki. Information was originally recorded and collected for medical purposes; therefore, consent was waived by the institutional review boards of the collaborating institutions.

### Participants

Five academic sites with designated inpatient and outpatient facilities for treatment of AML contributed to this study under the coordination of the FHCRC. The other 4 collaborating sites were Cleveland Clinic, Cleveland, Ohio; Massachusetts General Hospital, Boston; Stanford University, Stanford, California; and the University of Utah, Salt Lake City. Inclusion criteria were (1) age 20 years or older, (2) newly diagnosed non-M3 AML, and (3) initial treatment given during the study period defined herein. Patients receiving palliative care only were excluded from the data set. The different regimens used to treat these patients were classified as low, intermediate, or high intensity as indicated in eTable 1 in the Supplement.

### Outcome

Death within 1 year from initial therapy was the outcome of interest because it includes both early deaths due to regimen-related toxic effects or to lack of response and/or relapse of AML,^14^ as well as later deaths following treatment of relapse or refractory disease. Death within 8 weeks was analyzed as a secondary end point. Survival data were not known to those investigators who collected information on the potential predictors. Predictors, sample size, and missing data are described in detail in the eMethods in the Supplement.

### Statistical Analysis

The distribution of death according to time is described in eTable 2 of the Supplement. All 27 comorbidities and 8 covariates were tested in univariate analysis for their impact on 1-year mortality. Factors associated with 1-year mortality at *P* < .10 were used to construct a multivariate Cox proportional hazards model in which the impact of each comorbidity or covariate was adjusted for that of all others. To develop both a new AML-specific comorbidity index and a composite multirisk factor model, we used adjusted hazard ratio (HR) estimates for 1-year mortality from the multivariate model. The adjusted HRs were converted into integer weights according to our previously established criteria^10^: adjusted HRs of 1.2 or less were dropped from consideration, adjusted HRs of 1.3 to 1.9 were converted into a weight of 1.0, and adjusted HRs of 2.0 to 2.9 were converted into a weight of 2.0. None of the components had HRs greater than 2.9. The AML comorbidity index (AML-CI) was the sum of comorbidity integer weights. The augmented HCT-CI was the sum of HCT-CI comorbidities with the addition of weights for biomarker laboratory values (ie, albumin level, platelet counts, and lactate dehydrogenase [LDH]) level. The AML composite model (AML-CM) was the sum of integer weights of the augmented HCT-CI, with the addition of age groups and cytogenetic/molecular risk groups.

### Model Validation

Each risk model was validated in an independent set of 367 patients (eTable 3 of the Supplement compares the training and validation groups). We assessed the performances of the new AML-CI, the HCT-CI, the augmented HCT-CI, age groups, cytogenetic/molecular risk groups, and the AML-CM by computing the C statistic^17^ for a continuous predictor associated with time to death over 1 year. This can be interpreted as the probability that over many random pairs the patient with the shorter survival would have the worse score for the various potential predictors listed in the previous subsection. For binary outcomes (event times within 1 year and within 2 months), we computed the area under receiver operating characteristic curves (AUC). A value of 1.0 indicates perfect predictive discrimination, whereas a value of 0.5 indicates no ability to discriminate. A small number of patients censored prior to 2 months or 1 year were excluded from this calculation. Standard deviations of the C statistics and AUCs were estimated from 50 bootstrap samples. Statistical significance was determined by paired *t* test from the 50 bootstrap samples. Kaplan-Meier curves for survival were computed for risk groups defined by the different indices.

### Risk Groups

We defined risk groups for the 4 indices to represent approximate quartiles. Age and cytogenetic risk groups were grouped according to the weight assigned in the composite model.

## Results

### Participant Characteristics

The median age of patients was 60 years (range, 20-89 years). Overall, 605 patients (55%) were male, and 495 (45%) were female. eTable 4 in the Supplement shows demographic and disease characteristics for all patients as well as per site. The most obvious difference among sites was in the proportion of patients given high-, intermediate-, or low-intensity initial therapies (eTable 4 in the Supplement). The numbers of participants with missing data per each covariate are listed in eTables 5 and 6 in the Supplement. In a separate analysis, there was no evidence that missing vs nonmissing status was associated with outcome for any of the covariates (data are not shown). All 1100 participants had complete outcome data. As expected in a random assignment model, the training and validation sets appropriately had similar characteristics as well as 1-year mortality rate (eTable 3 in the Supplement).

### Model Development and Specification

eTable 2 in the Supplement indicates that 679 of the 1100 patients (62%) died, with 379 of the deaths (65%) occurring in the first year after start of initial therapy. Unadjusted associations between individual comorbidities and other covariates and 1-year posttreatment mortality are shown in eTable 7 in the Supplement. Cardiac comorbidity, diabetes, hepatic comorbidity, infection, peptic ulcer disease, renal comorbidity, prior malignant neoplasm, heart valve disease, hyperlipidemia, hypertension, hypoalbuminemia (albumin level <3.5 g/dL), thrombocytopenia (platelet count of <20 × 10^3^ cells/μL), and high LDH level (>200 U/L) values met the predetermined significance level (*P* < .10) in univariate analyses to be considered in multivariate models. In the multivariate analysis, 9 comorbidities met the predetermined cutoffs (HR >1.2) for adjusted HRs to be assigned a score for the AML-CI (Table 1). (To convert albumin to grams per liter, multiply by 10; to convert LDH to microkatals per liter, multiply by 0.0167). Adjusted HRs were then converted into scores where cardiac, hepatic dysfunction, infection, peptic ulcer, heart valve disease, albumin value less than 3.5 g/dL, platelet count less than 20 × 10^3^ cells/μL, and LDH values greater than 200 to 1000 U/L were each assigned a score of 1.0 (HR, 1.3-1.9), while an LDH value greater than 1000 U/L was assigned a score of 2.0 (HR, 2.0–2.9). Those 9 comorbidities constituted the new AML-CI.

**Table 1.** Multivariate Analysis of Associations Between Individual Comorbidities and Other Covariates With Post–Initial Therapy Mortality (288 Deaths Over 1 Year): Hazard Ratios (HRs) and Corresponding Scores for the AML-CI

Comorbidities	HR (95% CI)	Assigned Score for AML-CI	*P* Value
Cardiac	1.6 (1.2-2.3)	1	.05
Diabetes	1.1 (0.9-2.8)	0	.71
Hepatic	1.3 (1.0-1.8)	1	.04
Infection	1.3 (0.9-1.8)	1	.12
Peptic ulcer	1.6 (0.9-2.7)	1	.11
Renal			
Mild	1.1 (0.7-1.6)	0	.71
Moderate/severe	1.0 (0.6-1.5)	0	.84
Prior malignant neoplasm	1.2 (0.9-1.6)	0	.20
Heart valve disease	1.5 (0.9-2.8)	1	.16
Hyperlipidemia	0.9 (0.7-1.2)	0	.58
Hypertension	1.1 (0.8-1.4)	0	.66
Albumin level, g/dL			
<4.0-3.5	1.2 (0.8-1.6)	0	.43
<3.5-3.0	1.3 (0.9-1.8)	1	.20
<3.0	1.6 (1.0-2.4)		.04
Platelet count, ×10^3^ μL			
<100-50	1.1 (0.8-1.5)	0	.75
<50-20	1.0 (0.8-1.5)	0	.78
<20	1.3 (0.9-2.0)	1	.15
LDH level, U/L			
>200-500	1.7 (1.2-2.5)	1	.004
>500-1000	1.8 (1.1-2.7)	1	.01
>1000	2.2 (1.4-3.5)	2	.001
Sex			
Male	1.1 (0.8-1.4)	0	.68
Female	1 [Reference]	0	NA
Age, y			
0-49	1 [Reference]	0	NA
50-59	1.8 (1.2-2.7)	1	.007
60-69	2.0 (1.3-3.0)	2	.001
≥70	2.5 (1.5-4.0)		<.001
Cytogenetic/molecular risks			
Favorable	1 [Reference]	0	NA
Intermediate	1.8 (1.2-2.8)	1	.009
Adverse	2.8 (1.9-4.3)	2	<.001
Initial regimen intensity			
Low	1.6 (1.1-2.3)	NA	.008
Intermediate	1 [Reference]	NA	NA
High	1.2 (0.9-1.8)	NA	.25

Abbreviations: AML-CI, acute myeloid leukemia-specific comorbidity index; LDH, lactate dehydrogenase; NA, not applicable.

SI conversion factors: To convert albumin to grams per liter, multiply by 10; to convert LDH to microkatals per liter, multiply by 0.0167.

We compared the performance of the new AML-CI with that of the original HCT-CI.^10^ In addition, we constructed an augmented HCT-CI that is composed of the 17 comorbidities defining the original HCT-CI together with the 3 new independently significant comorbidities (hypoalbuminemia, thrombocytopenia, and high LDH values). eTable 8 in the Supplement summarizes these models.

Sex, age, European Leukemia Network (ELN) cytogenetic/molecular risk groups,^19^ and initial regimen intensity entered multivariate models. Sex did not meet criteria for score assignment, and regimen intensity was omitted from score assignment given its relation to patient baseline characteristics. For the purpose of developing a composite risk model, adjusted HRs for older age and cytogenetic/molecular risk groups were then converted into scores in which age of 50 to 59 years was assigned a score of 1.0 (HR, 1.8 vs age <50 years) and age of 60 years or older a score of 2.0 (HR, 2.0-2.5), intermediate cytogenetic/molecular risk group a score of 1 (HR, 1.8 vs favorable risk) and adverse cytogenetic/molecular risk group a score of 2 (HR, 2.8) (Table 1).

### Model Validation and Performance

In the validation set, the augmented HCT-CI had higher discriminative capacity for prediction of mortality compared with AML-CI as evaluated by C statistic (*P* = .004) and AUC (*P* = .01) (Table 2; eTable 9 of the Supplement). Age and cytogenetic/molecular risk groups were also valid in predicting 1-year and 8-week mortality (Table 2; eTable 9 of the Supplement); when both were added to the AML-CI they together yielded an AUC of 0.73 for 1-year mortality.

**Table 2.** Comparisons of the Performance of Risk Factors and Indices in Validation Set of 367 (148 Deaths)

Risk Factor	Components	C Statistic^a^ (SD^d^) for 1-y Mortality		True AUC^b^ (SD) for 1-y Mortality		True AUC^c^ (SD) for 8-wk Mortality	
		No.	(SD)	No.	(SD)	No.	(SD)
AML-CI	Cardiac, hepatic dysfunction, infection, peptic ulcer, heart valve disease, albumin level <3.5 g/dL, platelet count <20 × 10^3^ cells/μL, LDH level 200-1000 U/L, LDH level >1000 U/L	314	0.596 (0.019)	297	0.606 (0.039)	305	0.659 (0.043)
Original HCT-CI	17 covariates as previously described^10^	352	0.649 (0.025)	326	0.674 (0.028)	339	0.684 (0.042)
Augmented HCT-CI	Original HCT-CI + albumin level <3.5 g/dL, platelet count <20 × 10^3^ cells/μL, LDH level 200-1000 U/L, and LDH level >1000 U/L	305	0.664 (0.023)	289	0.687 (0.035)	296	0.721 (0.046)
Age (groups)	0-49 (score 0) vs 50-59 (score 1) vs ≥60 y (score 2)	367	0.640 (0.020)	340	0.682 (0.029)	354	0.640 (0.040)
Cytogenetic/molecular risks (groups)	ELN Favorable (score 0) vs intermediate (score 1) vs adverse (score 2)	350	0.614 (0.020)	324	0.654 (0.023)	337	0.597 (0.042)
AML-CM	Augmented HCT-CI + age + cytogenetic/molecular risks	292	0.719 (0.022)	277	0.758 (0.030)	283	0.776 (0.035)
KPS (groups)	100%-85% vs 80%-75% vs ≤70%-20%	291	0.619 (0.027)	266	0.646 (0.035)	279	0.676 (0.048)

Abbreviations: AML-CI, acute myeloid leukemia–comorbidity index; AML-CM, acute myeloid leukemia–composite model; AUC, area under the curve; HCT-CI, hematopoietic cell transplantation–comorbidity index; KPS, Karnofsky performance status; LDH, lactate dehydrogenase.

SI conversion factors: To convert albumin to grams per liter, multiply by 10; to convert LDH to microkatals per liter, multiply by 0.0167.

^a^ C statistic computed with full range of index for 148 deaths within 1 y.

^b^ AUC for 148 deaths within 1 y (excluding 27 patients censored before 1 y).

^c^ AUC for 45 deaths within 8 weeks (excluding 13 patients censored before 8 weeks).

^d^ Standard deviation estimated from 50 bootstrap simulations.

When age and cytogenetic/molecular risk groups were added to the augmented HCT-CI to form the AML-CM, this new composite model had a C statistic of 0.72 and AUC of 0.76 for 1-year mortality, and an AUC of 0.78 for 8-week mortality; all exceeded those of individual risk factors or models including performance status (Table 2; eTable 9 of the Supplement). For 1-year mortality, both the C-statistic estimate (SD) of 0.72 (0.02) (*P* < .001) and AUC of 0.76 (0.03) (*P* < .001) of the AML-CM were statistically significantly higher compared with those (C-statistic estimate of 0.66 [0.02] and AUC of 0.69 [0.04], respectively) of the augmented HCT-CI. The AML-CM also outperformed the Karnofsky performance status (KPS) (C statistic values for 1-year mortality of 0.72 vs 0.62). Visual representations of the higher C statistic and AUC values and hence better discriminative ability afforded by the AML-CM compared with age, KPS, ELN cytogenetic/molecular risk, the HCT-CI, the AML-CI, and the augmented HCT-CI can be found in the Figure and the eFigure in the Supplement, while Table 2 and eTable 9 of the Supplement show this in tabular form. Components of the HCT-CI, the augmented HCT-CI and the AML-CM are described in eTable 8 in the Supplement.

**Figure.**
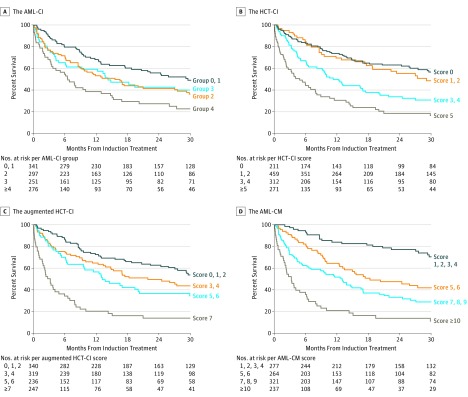
Kaplan-Meier Estimates of Survival Kaplan-Meier estimates of survival. A, Estimated survival stratified according to the acute myeloid leukemia-comorbidity index (AML-CI). B, Estimated survival stratified according to the hematopoietic cell transplantation-comorbidity index (HCT-CI). C, Estimated survival stratified according to the augmented HCT-CI. D, Estimated survival stratified according to the AML-composite model (AML-CM).

The higher performance of the AML-CM persisted in comparison with KPS. The AML-CM provided better discrimination of mortality rates compared with all other risk factors, as illustrated in the Figure and in the eFigure and eTable 9 of the Supplement. Components of the augmented HCT-CI and the AML-CM are described in eTable 8 in the Supplement.

## Discussion

Patients with AML have a relatively high early mortality rate of 37.5% at 8 weeks^16^ as well as a high overall mortality rate of 76% at 3 years.^20^ These rates are even higher among patients 65 years or older.^20^ Treatment of AML is also extremely costly and disruptive to patients and families given the lengthy and frequent hospitalizations.^21^ Furthermore, available therapies for patients with AML vary widely in their complexity and intensity. Therefore, the ability of physicians to make accurate predictions about the likely outcome of initial AML therapy is important in arriving at decisions to give conventional therapy of varying intensity, investigational treatment, or supportive care only. Until now, decisions about the choice of therapy have largely been based on age (eg, ≥65 years or <65 years), even though commonplace observation suggests some older patients can sometimes be healthier than some younger ones. Performance status often influences decisions and is frequently used to define the vague notion of “unfit for intensive therapy.” However, formal evaluation of comorbidities has played a small role in decisions about initial therapy. Indeed, comorbidities are a principal determinant of morbidity and mortality after treatment of older patients with other hematological malignant neoplasms^22^ and are independent of functional status.^23^ We have previously demonstrated that an HCT-CI is an important predictor of outcome after HCT and, in particular, is more important than age in this regard. However, the value of the HCT-CI in a general AML population receiving initial therapy is unknown.

In the current study, we have shown and validated that comorbidities have a significant impact on early and 1-year mortality among patients newly diagnosed with AML undergoing upfront therapies. In addition, we have demonstrated that an augmented HCT-CI performs better than either an AML-CI or the original HCT-CI in predicting early and late mortality. The HCT-CI has been in practice since 2005, making it quite familiar to and easily applicable by physicians. Most notably, when we incorporated comorbidities together with age and cytogenetic/molecular risks per the ELN classification into a composite model (AML-CM), this risk model performed better than each of the risk components alone (Table 2, Figure; eFigure in the Supplement). Just as a comorbidity assessment—using the HCT-CI before allogeneic HCT has had a major impact on the decision to proceed to HCT—we believe use of the AML-CM could inform decisions as to whether patients with newly diagnosed AML should receive more intensive or less intensive therapies for their disease.

On the one hand, the inability to develop a comorbidity index specific to AML that outperforms the original HCT-CI matches our experience in another setting, the prediction of graft-vs-host disease after allogeneic HCT.^24^ On the other hand, the augmentation of the performance of the HCT-CI by adding albumin values, as a marker of nutritional or inflammatory status, and platelet counts, as a marker of bone marrow health, agrees with our previous findings in the setting of allogeneic HCT.^25^ Currently, we are prospectively investigating the value of adding data on pulmonary function tests to comorbidity assessment before initial therapy for AML. The strong impact of cytogenetic/molecular risks on mortality is not a surprise.^19^ Why increasing age continues to have a significantly independent impact on mortality after accounting for comorbidities is unclear. One explanation could be the acquisition of additional adverse molecular AML markers with aging.^26^ Alternatively, aging could be a surrogate for other forms of health limitations, for example, functional, cognitive, or social.^4^ Our current efforts are directed toward quantification and understanding of such aging-related risks (NCT01929408). Finally, we did not incorporate regimen intensity, albeit predictive of mortality, in our model since this is the decision that we plan to improve based on the AML-CM scores.

### Limitations

Limitations of the current study include the retrospective nature of data collection, potentially leading to failure to capture important data that might not have been recorded. However, the frequent use of laboratory data, most of which are consistently available in databases, to define comorbidities likely reduced the possibility of missing comorbidities. Most notably, the collected data for components of the HCT-CI were almost complete. Moreover, missing data on a few other comorbidities or covariates did not exceed 10%, and we have no reason to believe that missing data from retrospective medical record review were systematically related to outcome or other predictive factors. We confirmed that missing vs nonmissing data were not associated with outcome in this study. Regarding reproducibility of comorbidity assessments, while we did not perform analyses on interrater (IRR) and test-retest reliabilities for the augmented HCT-CI, we used recently described methods for comorbidity evaluation that indicated an IRR greater than 0.90 by weighted κ statistics.^18^ The use of data from multiple centers, as well as the inclusion of all patients receiving any form of AML-therapy, increases the generalizability of our findings. Previously, in the allogeneic HCT setting, the original HCT-CI was proven valid in published prospective studies,^27^ and an ongoing observational study (NCT01929408) seeks to prospectively validate the AML-CM in patients with newly diagnosed AML. The AML-CM was designed to primarily predict survival at 1 year. The 1-year end point is intuitively appealing, and incorporates both treatment-related mortality and inherent resistance to antileukemic therapy. In contrast, an earlier end point may have lent undue significance to the former and a significantly later end point undue significance to the latter consideration. Nevertheless, the AML-CM strongly predicted earlier mortality at 8 weeks after initial therapy. Finally, although many patients receiving therapies of differing intensities were included, the intensity of therapy was not randomly assigned. We arbitrarily categorized those regimens based on feedback from all study collaborators into 3 levels of intensity. However, we did not incorporate regimen intensity, albeit predictive of mortality, in our model since what constitutes high-, intermediate-, and low-intensity therapy is arguable, and we are in the process of developing objective means to assess regimen intensity and to formally assess the benefits of regimens, thus defined, according to AML-CM scores.

### Model Applications and Benefits

The previous limitations notwithstanding, our results have significant clinical and scientific applications. Currently, decisions about initial therapy for newly diagnosed AML are based largely on age and an often *subjective* assessment of a patient’s fitness. The use of the AML-CM by community internists will allow *objective* identification of those older patients with AML, who have traditionally been offered palliative care but who might be better served by referral to receive either conventional or investigational AML therapy. Consequently, some patients with AML who currently may not be referred for specific treatment will be referred in the future, leading to improved survival of patients nationwide. Conversely, the AML-CM could identify patients so unlikely to benefit from AML therapy that they could be spared the various risks and toxic effects of such therapies. In the future, the AML-CM might also be used to identify which patients would benefit more from intensive chemotherapy and which will be better served by targeted, less-intensive therapies, and we are organizing a national effort to examine this question. Whether the AML-CM calculated at diagnosis of AML can be later used to decide on allogeneic HCT vs non-HCT therapies is currently being studied in an ongoing observational study (NCT01929408). The AML-CM may also allow for more objective comparisons of outcomes with different therapies or with the same treatment at different centers. Finally, the identification of patients with high comorbidity burden at diagnosis will provide impetus for interventions to reduce the effects of these comorbidities before or simultaneously with the administration of initial AML therapy.

## Conclusions

We have developed a novel AML composite model that allows us to balance the effect of age with the effects of the patients’ overall health, as assessed by comorbidities, and the aggressiveness of their AML, as assessed by cytogenetic/molecular features. The AML-CM outperforms age and KPS in predicting early and late mortality and hence could replace these conventional covariates when making treatment decisions or comparing trial results. This model could prove useful to the US Food and Drug Administration when monitoring clinical trials to ensure adequate representation of high-risk patients in these trials and, hence, generalizability of trial results to the whole AML population. To facilitate the future use of this model, we are constructing a web-based calculator (http://www.AMLCompositeModel.org).

## Notes

Supplement.
eMaterials and Methods

eReferences
**eTable 1.** Initial Therapy Regimens as Stratified into Low, Intermediate, and High Intensity **eTable 2.** Distribution of Mortality Events (n = 679) According to Time among Patients of the Training Set (n = 733) **eTable 3.** Comparison of characteristics of patients within the training and validation sets **eTable 4.** Patient Characteristics at Diagnosis of Acute Myeloid Leukemia for All Patients as well as and per Institution **eTable 5.** Distribution and Classification of HCT-CI and Other Comorbidities **eTable 6.** Distribution and Classification of Other Covariates **eTable 7.** Univariate Analysis of Associations between Individual Comorbidities and Other Covariates with Post-Initial Therapy Mortality (288 Deaths Over 1 Year) **eTable 8.** Definitions of the HCT-CI, augmented HCT-CI, and the AML-CM **eTable 9.** Comparisons of Kaplan-Meier Survival Rates per Different Risk Factors and Models **eTable 10.** Power Calculations for Sample Size **eFigure.** Kaplan-Meier Estimates of Survival
