# Supplementary material for: Development and Validation of a Novel Acute Myeloid Leukemia–Composite Model to Estimate Risks of Mortality
Source: JAMA Oncol. 2017 Sep 7;3(12):1675–82. doi: 10.1001/jamaoncol.2017.2714 (PMC5824273; doi:10.1001/jamaoncol.2017.2714)
Supplement: Supplement. — eMaterials and Methods eReferences eTable 1. Initial Therapy Regimens as Stratified into Low, Intermediate, and High Intensity eTable 2. Distribution of Mortality Events (n = 679) According to Time among Patients of the Training Set (n = 733) eTable 3. Comparison of characteristics of patients within the training and validation sets eTable 4. Patient Characteristics at Diagnosis of Acute Myeloid Leukemia for All Patients as well as and per Institution eTable 5. Distribution and Classification of HCT-CI and Other Comorbidities eTable 6. Distribution and Classification of Other Covariates eTable 7. Univariate Analysis of Associations between Individual Comorbidities and Other Covariates with Post-Initial Therapy Mortality (288 Deaths Over 1 Year) eTable 8. Definitions of the HCT-CI, augmented HCT-CI, and the AML-CM eTable 9. Comparisons of Kaplan-Meier Survival Rates per Different Risk Factors and Models eTable 10. Power Calculations for Sample Size eFigure. Kaplan-Meier Estimates of Survival [file jamaoncol-3-1675-s001.pdf]

# Supplementary Online Content

Sorror ML, Storer BE, Fathi AT, et al. Development and validation of a novel acute myeloid leukemia–composite model to estimate risks of mortality. *JAMA Oncol*. Published online September 7, 2017. doi:10.1001/jamaoncol.2017.2714

**eMaterials and Methods**

**eReferences**

**eTable 1.** Initial Therapy Regimens as Stratified into Low, Intermediate, and High Intensity

**eTable 2.** Distribution of Mortality Events (n=679) According to Time among Patients of the Training Set (n=733)

**eTable 3.** Comparison of characteristics of patients within the training and validation sets

**eTable 4.** Patient Characteristics at Diagnosis of Acute Myeloid Leukemia for All Patients as well as and per Institution

**eTable 5.** Distribution and Classification of HCT-CI and Other Comorbidities

**eTable 6.** Distribution and Classification of Other Covariates

**eTable 7.** Univariate Analysis of Associations between Individual Comorbidities and Other Covariates with Post-Initial Therapy Mortality (288 Deaths Over 1 Year)

**eTable 8.** Definitions of the HCT-CI, augmented HCT-CI, and the AML-CM

**eTable 9.** Comparisons of Kaplan Meier Survival Rates per Different Risk Factors and Models

**eTable 10.** Power Calculations for Sample Size

**eFigure.** Kaplan Meier Estimates of Survival

This supplementary material has been provided by the authors to give readers additional information about their work.

© 2017 Sorror ML, et al. *JAMA Oncology.*

**SUPPLEMENTAL ONLINE-ONLY MATERIAL for SORROR et al.,**
**" The Acute Myeloid Leukemia – Composite Model: A New Tool for Risk-Assessment"**

**List of ONLINE-ONLY Items**


1. Supplemental eMaterials and Methods (including eReferences)
   a. Predictors: Comorbidities and Other Covariates
   b. Sample Size
   c. Missing Data
2. Supplemental Results
   a. Use of the AML-CM in Describing Outcomes of Patients of ≥65 years of age.
3. Supplemental eTables
   a. eTable 1. Initial Therapy Regimens as Stratified into Low, Intermediate, and High Intensity.
   b. eTable 2: Distribution of Mortality Events (n=679) According to Time among Patients of the Training Set (n=733).
   c. eTable 3: Comparison of characteristics of patients within the training and validation sets.
   d. eTable 4: Patient Characteristics at Diagnosis of Acute Myeloid Leukemia for All Patients as well as and per Institution.
   e. eTable 5: Distribution and Classification of HCT-CI and Other Comorbidities.
   f. eTable 6: Distribution and Classification of Other Covariates.
   g. eTable 7: Univariate Analysis of Associations between Individual Comorbidities and Other Covariates with Post-Initial Therapy Mortality (288 Deaths Over 1 Year).
   h. eTable 8: Definitions of the HCT-CI, augmented HCT-CI, and the AML-CM
   i. eTable 9: Comparisons of Kaplan Meier Survival Rates per Different Risk Factors and Models.
   j. eTable 10: Power Calculations for Sample Size.
4. Supplemental eFigures
   a. eFigure 1: Kaplan Meier Estimates of Survival.


© 2017 Sorror ML, et al. *JAMA Oncology.*

**SUPPLEMENTAL MATERIALS AND METHODS (eMaterials and Methods)**

**Predictors:** Comorbidities and Other Covariates

Comorbidities were evaluated per the definitions of comorbidities for the HCT-CI;[1,2] with the exception of renal comorbidities that were defined by creatinine clearance (mild = <60-46, moderate = <46-31, and severe = <31) when available and by serum creatinine when creatinine clearance was not available. Data on additional comorbidities not included in the HCT-ICI were collected including hyperlipidemia, hypertension, coagulopathy, gastroesophageal reflux disease, hypothyroidism, hypoalbuminemia, elevated lactate dehydrogenase (LDH), thrombocytopenia, neutropenia, anemia, and smoking history. These additional comorbidities were defined as follows:

*Hyperlipidemia:* Diagnosis of hyperlipidemia at any time in the patient past medical history. Patient had to have been on a specific treatment for hyperlipidemia for at least 4 consecutive weeks before start of induction therapy.

*Hypertension:* Diagnosis of hypertension at any time in the patient past medical history. Patient had to have been on a specific treatment for hypertension for at least 4 consecutive weeks before start of induction therapy.

*Coagulopathy:* Diagnosis of deep venous thrombosis or pulmonary embolism at any time in the patient past medical history.

*Gastroesophageal reflux disease (GERD):* Diagnosis of GERD at any time in the patient past medical history. Patient had to have been on a specific treatment for GERD for at least 4 consecutive weeks before start of induction therapy.

© 2017 Sorror ML, et al. *JAMA Oncology.*

*Hypothyroidism:* Diagnosis of hypothyroidism at any time in the patient past medical history. Patient had to have been on a specific treatment for hypothyroidism for at least 4 consecutive weeks before start of induction therapy.

*Hypoalbuminemia:* We evaluated the closest value of albumin before start of induction therapy. We categorized albumin values into three categories: 3.5-<4, 3-<3.5, and <3 g/dL.

*Elevated lactate dehydrogenase (LDH):* We evaluated the closest value of LDH before start of induction therapy. We categorized LDH values into three categories: >200-500, >500-1000, and >1000 U/L.

*Thrombocytopenia:* We evaluated the closest value of platelet count before start of induction therapy. We categorized platelet count values into three categories: <100,000-50,000, <50,000-20,000, and <20,000 cells/μL.

*Neutropenia:* We evaluated the closest value of neutrophil count before start of induction therapy. We categorized neutrophil count values into three categories: <1,500-1,000, <1,000-500, and <500 cells/μL.

*Anemia:* We evaluated the closest value of hemoglobin before start of induction therapy. We categorized hemoglobin values into three categories: <10-9, <9-8, and <8 g/dL.

*Smoking:* We evaluated smoking status before start of induction therapy. We categorized smoking status into three categories: current (within 3 months before start of

© 2017 Sorror ML, et al. *JAMA Oncology.*

induction therapy), former (cessation of smoking more than 3 months before start of induction therapy), and never.

Induction treatment regimens were categorized into 3 levels of intensity: low, intermediate, and high (eTable 1). Cytogenetic-molecular risks were categorized into favorable 19%, intermediate 38%, and adverse 43% following the European Leukemia Network (ELN) classification.[3]

Other Covariates are described in Supplemental eTable 6.

**Sample Size**

Sample size was determined based on the total number of patients seen at each of the five institutions during the study, conducted from 2008-2012. Supplemental eTable 10 gives the minimal increment in the 1-year risk of mortality detectable with at least 80% power at the 2-sided 0.05 level of significance in the training set of 733 patients. Since there is almost no loss to follow-up for the primary endpoint, the power calculations are based on a binomial assumption; the actual analysis based on Cox regression should provide somewhat higher power. Supplemental eTable 10 demonstrates sufficient power to identify a minimal increase in risk for a risk factor of specified prevalence compared to the remainder of the cohort. As noted in Supplemental eTable 2 there was an all-cause mortality rate of 40% (428/1100) in the first year.

**Missing Data**

Data collected from all 1100 pateints were used in the study analyses. Supplemental eTables 5 and 6 describe the distribution of all covariates, comorbidities as well other covariates, and describe the number and percentage of missing data per covariate. The components of the

© 2017 Sorror ML, et al. *JAMA Oncology.*

HCT-CI were almost complete, and the HCT-CI could be computed for all patients except for a few (n=41), who were missing body mass index that defines obesity. Univariate analyses of risk factors excluded patients with missing data for the risk factor. In the multivariate analysis performed within the training set, missing value indicator variables were included for albumin, platelets, LDH, and cytogenetic risk group. In the validation set, we used complete data analysis in computing the various indices for comparisons of discriminative capacity.

## SUPPLEMENTAL RESULTS (eResults)

### Use of the AML-CM in Describing Outcomes of Patients of ≥65 years of age

We have looked at data on patients 60-75 years old within the validation set. Those patients constituted about 50% of the validation set population. The AUC for 1-year mortality was 0.68. The reduced AUC compared to the general population of AML is expected since this group is homogenous with respect to an important component of the AML-CM, alas age.

Per the AML-CM four risk groups, 1-year survival among patients 60-75 years old were; for those with scores 1-4, 86%; scores 5-6, 50%; scores 7-9, 46%; scores ≥10, 23%.

These results suggest that for older patients, AML-CM categorization into 3 risk groups with AML-CM scores of 1-4, 5-9, and ≥10 is more appropriate for decision-making given similar outcomes between those with scores 5-6 and 7-9.

Patients with scores 1-4 could benefit from intensive standard treatment combinations. Patients with scores ≥10 would benefit from being enrolled in novel clinical trials using novel targeted therapies to improve their outcomes. The concurrent use of palliative care might be warranted in this patient population. Patients with scores of 5-9 could be good candidates for randomized controlled trial comparing intensive versus less-intensive induction therapies for AML.

© 2017 Sorror ML, et al. *JAMA Oncology.*

## Supplemental References


1. Sorror M. How I assess comorbidities prior to hematopoietic cell transplantation. *Blood.* 2013;121(15):2854-2863.
2. Sorror ML, Maris MB, Storb R, et al. Hematopoietic cell transplantation (HCT)-specific comorbidity index: a new tool for risk assessment before allogeneic HCT. *Blood.* 2005;106(8):2912-2919.
3. Dohner H, Estey EH, Amadori S, et al. Diagnosis and management of acute myeloid leukemia in adults: recommendations from an international expert panel, on behalf of the European LeukemiaNet (Review). *Blood.* 2010;115(3):453-474.


© 2017 Sorror ML, et al. *JAMA Oncology.*

**eTable 1. Initial Therapy Regimens as Stratified into Low, Intermediate, and High Intensity**

| | | |
|---|---|---|
| **Low Intensity** | | |
| Regimen | | Patients |
| Main agent | Added agents | N |
| **LDAC** | | **44** |
| | Alone | 15 |
| | + Clofarabine | 11 |
| | + Tosedostat | 11 |
| | + Barasertib | 2 |
| | + Lintuzumab | 2 |
| | + Rigosertinib | 2 |
| | + Azacitedine | 1 |
| **Azacitidine** | | **67** |
| | Alone | 51 |
| | + Gemtuzumab Ozogamicin | 10 |
| | + Gemtuzumab Ozogamicin + vorinostat | 3 |
| | + Decitabine | 1 |
| | + Lenalidomide | 1 |
| | + Vorinostat | 1 |
| **Decitabine** | | **40** |
| | Alone | 33 |
| | + Bortezomib | 6 |
| | + Gemtuzumab Ozogamicin | 1 |
| **Bendamustine + Idarubicin** | | **30** |
| **Clofarabine** | **Alone** | **12** |
| **Gemtuzumab Ozogamicin + vorinostat** | | **16** |
| **Others** | | **19** |
| **Moderate Intensity** | | |
| **"7+3"** | | **578** |
| | Alone | 490 |
| | + Gemtuzumab Ozogamicin | 28 |
| | + Bortezomib | 12 |
| | + Etopsoside | 12 |
| | + Midostaurine vs placebo | 9 |
| | + Decitabine | 5 |
| | + Cladribine | 4 |
| | + Sorafenib | 4 |
| | + Dasatinib | 3 |
| | + Panobinostat | 3 |
| | + Plerixafor | 3 |
| | + Pravastatin | 2 |
| | + Imatinib | 2 |
| | + Vincristine, Dexamethasone | 1 |
| **Cytarabine (100 mg/m$^2$/dose)** | | **45** |
| | + Mitoxantrone | 37 |

© 2017 Sorror ML, et al. *JAMA Oncology.*

| | | |
|---|---|---|
| | + Amonafide | 5 |
| | + Clofarabine | 1 |
| | + Methotrexate | 1 |
| | + Pravastatin | 1 |
| **“4+3”** | | **40** |
| | Alone | 37 |
| | + Dasatinib | 3 |
| **Others** | | **12** |
| | **High Intensity** | |
| **Cytarabine (≥1 g/m$^2$/dose)** | | **199** |
| | Alone | 2 |
| | + Purine analog (fludarabine, cladrabine, or clofarabine) | 116 |
| | + Idarubicin | 66 |
| | + Others | 15 |

LDAC indicates low-dose cytarabine; “7+3” or “4+3”, Cytarabine, + Daunorubicin, Doxorubicin, Idarubicin or Mitoxantrone.

© 2017 Sorror ML, et al. *JAMA Oncology.*

**eTable 2. Distribution of mortality events (n=679) according to time among all patients (n=1100)**

| Interval, months | Deaths | Cumulative |
|---|---|---|
| 02 | 135 | 135 |
| >2–4 | 71 | 206 |
| >4–6 | 67 | 273 |
| >6–8 | 56 | 329 |
| >8–10 | 56 | 385 |
| >10–12 | 53 | 438 |
| >12–18 | 100 | 538 |
| >18–24 | 45 | 583 |
| >24–36 | 55 | 638 |
| >36 | 41 | 679 |

© 2017 Sorror ML, et al. *JAMA Oncology.*

**eTable 3. Comparison of Characteristics and Outcome of Patients within the Training and Validation Sets**

| Patient Characteristics | All patients (n=1100) | Training (n-733) | Validation (n=367) |
|---|---|---|---|
| Age, median (range) | 60 (20–89) | 60 (20–88) | 59 (20–89) |
| | % | | |
| Sex | | | |
| Female | 45 | 45 | 45 |
| Male | 55 | 55 | 55 |
| Race | | | |
| Caucasian | 80 | 80 | 81 |
| Other | 20 | 20 | 19 |
| Cytogenetic risk, % | | | |
| Favorable | 19 | 20 | 17 |
| Intermediate | 38 | 40 | 36 |
| High | 43 | 41 | 47 |
| Regimen Intensity, % | | | |
| Low | 20 | 20 | 20 |
| Intermediate | 62 | 63 | 60 |
| High | 19 | 18 | 20 |
| Outcome | | | |
| 1-year mortality | 39.5 | 39 | 40 |

© 2017 Sorror ML, et al. *JAMA Oncology.*

**eTable 4. Patient Characteristics at Diagnosis of Acute Myeloid Leukemia for All Patients as well as and per Institution.**

| | All patients (1100) | FHCRC (n=366) | Cleveland Clinic (n=216) | MGH (n=232) | Stanford University (n=198) | Utah University (n=88) |
|---|---|---|---|---|---|---|
| Age, median (range) | 60 (20-89) | 61 (20–84) | 59 (23–82) | 64 (20–89) | 55 (20–72) | 59 (20–88) |
| | % | | | | | |
| Sex | | | | | | |
| Female | 45 | 40 | 49 | 46 | 49 | 44 |
| Male | 55 | 60 | 52 | 54 | 52 | 56 |
| Race | | | | | | |
| Caucasian | 80 | 82 | 85 | 88 | 58 | 94 |
| Other | 20 | 18 | 15 | 12 | 42 | 6 |
| Cytogenetic/molecular risk | | | | | | |
| Favorable | 19 | 18 | 24 | 13 | 22 | 23 |
| Intermediate | 38 | 42 | 39 | 39 | 31 | 37 |
| Adverse | 43 | 41 | 37 | 49 | 47 | 40 |
| Regimen Intensity | | | | | | |
| Low | 20 | 33 | 4 | 25 | 5 | 25 |
| Intermediate | 62 | 31 | 93 | 72 | 70 | 69 |
| High | 19 | 37 | 3 | 4 | 25 | 7 |

FHCRC indicates Fred Hutchinson Cancer Research Center; MGH, Massachusetts General Hospital.

© 2017 Sorror ML, et al. *JAMA Oncology.*

**Supplemental eTable 5. Distribution and Classification of HCT-CI and Other Comorbidities (n=1100 patients)**

| Comorbidities | | | Prevalence of comorbidity | Grade of severity | | |
|---|---|---|---|---|---|---|
| | Missing data among patients | | | Mild | Moderate | Severe |
| | Number | % | % | % | % | % |
| Arrhythmia | 0 | 0 | 8 | – | – | – |
| Coronary Artery | 0 | 0 | 13 | – | – | – |
| Heart valve disease | 0 | 0 | 3 | – | – | – |
| Cerebrovascular | 0 | 0 | 4 | – | – | – |
| Inflammatory Bowel | 0 | 0 | 3 | – | – | – |
| Hepatic | 0 | 0 | 29 | 22 | 4 | 3 |
| Renal (serum creatinine)* | 0 | 0 | 19 | 16 | 2 | 1 |
| Pulmonary | 0 | 0 | 17 | 8 | 5 | 4 |
| Rheumatoid | 0 | 0 | 16 | – | – | – |
| Diabetes | 0 | 0 | 11 | – | – | – |
| Tumor | 0 | 0 | 21 | – | – | – |
| Peptic Ulcer | 0 | 0 | 3 | – | – | – |
| Psychiatric | 0 | 0 | 13 | – | – | – |
| Infection | 0 | 0 | 28 | – | – | – |
| Obesity | 41 | 3.7 | 13 | – | – | – |
| Renal (creatinine clearance)* | 195 | 17.7 | 19 | 9 | 7 | 3 |
| Hyperlipidemia | 0 | 0 | 27 | | | |
| HTN | 0 | 0 | 35 | | | |
| DVT | 0 | 0 | 4 | | | |
| GERD | 0 | 0 | 13 | | | |
| Hypothyroid | 0 | 0 | 11 | | | |
| Hyperthyroid | 88 | 8.0 | 1 | | | |
| Hypoalbuminemia | 65 | 5.9 | 72 | 31 | 24 | 17 |
| Elevated lactate dehydrogenase (LDH) | 114 | 10.4 | 80 | 44 | 21 | 15 |
| Thrombocytopenia | 38 | 3.5 | 70 | 27 | 29 | 14 |
| Neutropenia | 71 | 6.5 | 60 | 10 | 22 | 28 |
| Anemia | 39 | 3.5 | 67 | 26 | 24 | 17 |
| Smoking£ | 47 | 4.3 | 46 | | | |

In Blue are comorbidities included under the HCT-CI while in Red are other comorbidities

© 2017 Sorror ML, et al. *JAMA Oncology.*

*Both values were used to define renal comorbidity where preference was given to creatinine clearance when available or serum creatinine used if not.

£Smoking is categorized into never (50%), former (32%), current (14%).

Hypoalbuminemia was defined as a comorbidity if albumin value was <4 g/dL; mild if 3.5-<4; moderate, 3-<3.5; and severe, <3 g/dL.

Elevated LDH was defined as a comorbidity if LDH value was >200 U/L; mild if >200-500; moderate, >500-1000; and severe, >1000 U/L.

Thrombocytopenia was defined as a comorbidity if platelet value was <100,000 cells/μL; mild if <100,000-50,000; moderate, <50,000-20,000; and severe, <20,000 cells/μL

Neutropenia was defined as a comorbidity if neutrophil count value was <1,500 cells/μL; mild if <1,500-1,000; moderate, <1,000-500; and severe, <500 cells/μL.

Anemia was defined as a comorbidity if hemoglobin value was <10 g/dL; mild if <10-9; moderate, <9-8; and severe, <8 g/dL.

© 2017 Sorror ML, et al. *JAMA Oncology.*

**Supplemental eTable 6. Distribution and Classification of Other Study Covariates (n=1100 patients)**

| Covariate | Missing data among patients | | Categories | % of patients |
|---|---|---|---|---|
| | Number | % | | |
| Gender | 1 | 0.1 | Female | 45 |
| | | | Male | 55 |
| Age, years | 0 | 0 | 0–49 | 25 |
| | | | 50–59 | 23 |
| | | | 60–69 | 31 |
| | | | ≥70 | 20 |
| Race | 27 | 2.5 | White | 80 |
| | | | Asian | 5 |
| | | | Others | 15 |
| Cytogenetic/molecular risk | 52 | 4.7 | Favorable | 19 |
| | | | Intermediate | 38 |
| | | | Adverse | 43 |
| Initial regimen intensity | 2 | 0.2 | Low | 20 |
| | | | Intermediate | 62 |
| | | | High | 19 |
| WBC at diagnosis, X 1000 cells/μL | 20 | 1.8 | 0–5 | 45 |
| | | | >5–10 | 13 |
| | | | >10–25 | 14 |
| | | | >25–50 | 10 |
| | | | >50–100 | 10 |
| | | | >100 | 9 |
| Peripheral blast count at diagnosis, X 1000 cells/μL | 84 | 7.6 | 0–5 | 61 |
| | | | >5–10 | 7 |
| | | | >10–25 | 12 |
| | | | >25–50 | 7 |
| | | | >50–100 | 9 |
| | | | >100 | 4 |
| Marrow blasts at diagnosis, % | 107 | 9.7 | ≥ 20 | 19 |
| | | | >20–50 | 39 |
| | | | >50 | 43 |

© 2017 Sorror ML, et al. *JAMA Oncology.*

**eTable 7: Univariate Analysis of Associations between Individual Comorbidities and Other Covariates with Post-Initial Therapy Mortality (288 Deaths Over 1 Year)**

| **Comorbidities** | | HR | 95% confidence interval | P | Overall P |
|---|---|---|---|---|---|
| Arrhythmia | | 1.14 | (0.7–1.8) | 0.54 | |
| Cardiac | | 2.28 | (1.7–3.0) | <0.0001 | |
| Inflammatory Bowel | | 1.03 | (0.5–2.1) | 0.93 | |
| Diabetes | | 1.67 | (1.2–2.3) | 0.004 | |
| Cerebrovascular | | 1.37 | (0.8–2.4) | 0.27 | |
| Psychiatric | | 1.21 | (0.9–1.7) | 0.26 | |
| Hepatic | Mild | 1.47 | (1.1–1.9) | 0.003 | 0.004 |
| | Moderate/Severe | 1.44 | (1.0–2.2) | 0.08 | |
| Obesity | | 0.96 | (0.7–1.4) | 0.81 | |
| Infection | | 1.27 | (1.0–1.6) | 0.06 | |
| Rheumatologic | | 1.02 | (0.6–1.7) | 0.94 | |
| Peptic Ulcer | | 2.05 | (1.2–3.4) | 0.01 | |
| Renal | Mild | 1.38 | (1.0–1.8) | 0.03 | 0.06 |
| | Moderate/Severe | 1.44 | (0.8–2.6) | 0.22 | |
| Pulmonary | Mild | 1.20 | (0.8–1.8) | 0.35 | 0.20 |
| | Moderate/Severe | 1.32 | (0.9–1.9) | 0.11 | |
| Prior Malignancy | | 1.53 | (1.2–2.0) | 0.002 | |
| Heart Valve Disease | | 2.86 | (1.7–4.8) | 0.0006 | |
| Hyperlipidemia | | 1.58 | (1.2–2.0) | 0.0003 | |
| Hypertension | | 1.62 | (1.3–2.0) | <0.0001 | |
| Coagulopathy | | 0.98 | (0.5–1.7) | 0.94 | |
| GERD | | 1.13 | (0.8–1.6) | 0.46 | |
| Hypothyroid | | 1.17 | (0.8–1.7) | 0.42 | |
| Albumin, g/dL | <4–3.5 | 1.22 | (0.9–1.7) | 0.24 | 0.02 |
| | <3.5–3 | 1.42 | (1.0–2.0) | 0.04 | |
| | <3 | 1.73 | (1.2–2.5) | 0.003 | |
| Platelets, cells/μL | <100,000–50,000 | 1.19 | (0.9–1.6) | 0.27 | 0.08 |
| | <50,000–20,000 | 1.15 | (0.8–1.6) | 0.38 | |
| | <20,000 | 1.64 | (1.1–2.4) | 0.008 | |
| ANC, μL | <1,500–1,000 | 0.90 | (0.6–1.4) | 0.63 | |

© 2017 Sorror ML, et al. *JAMA Oncology.*

| | | | | | |
|---|---|---|---|---|---|
| | <1,000–500 | 1.11 | (0.8–1.5) | 0.50 | |
| | <500 | 0.78 | (0.6–1.1) | 0.11 | 0.20 |
| Hemoglobin, g/dL | <10–9 | 1.25 | (0.9–1.7) | 0.16 | |
| | <9–8 | 1.44 | (1.1–2.0) | 0.02 | |
| | <8 | 1.20 | (0.8–1.7) | 0.32 | 0.14 |
| LDH, U/L | >200–500 | 1.64 | (1.2–2.3) | 0.006 | |
| | >500–1000 | 1.67 | (1.1–2.5) | 0.01 | |
| | >1000 | 2.02 | (1.3–3.1) | 0.001 | 0.005 |
| Smoker | Former | 1.28 | (1.0–1.7) | 0.006 | |
| | Current | 1.06 | (0.7–1.5) | 0.75 | 0.19 |
| **Other Covariates** | | | | | |
| Gender | Female | 1.0 | — | — | |
| | Male | 1.25 | (1.0–1.6) | 0.06 | |
| Age, years | 0–49 | 1.0 | — | — | |
| | 50–59 | 1.81 | (1.2–2.7) | 0.003 | |
| | 60–69 | 2.42 | (1.7–3.5) | <0.0001 | <0.0001 |
| | ≥70 | 3.49 | (2.4–5.1) | <0.0001 | |
| Race | White | 1.0 | — | | |
| | Others | 0.93 | (0.7–1.2) | 0.61 | |
| Cytogenetic/molecular risks | Favorable | 1.0 | — | — | |
| | Intermediate | 1.82 | (1.25–2.5) | 0.006 | <0.0001 |
| | Adverse | 1.64 | (1.3–2.1) | 0.0002 | |
| Initial regimen intensity | Low | 1.93 | (1.5–2.5) | <0.0001 | |
| | Intermediate | 1.0 | — | — | <0.0001 |
| | High | 1.02 | (0.7–1.4) | 0.88 | |
| WBC, cells/μL | 0–5 | 1.0 | — | | |
| | >5–25 | 1.04 | (0.8–1.4) | 0.79 | |
| | >25–100 | 1.07 | (0.8–1.5) | 0.68 | 0.98 |
| | >100 | 1.04 | (0.7–1.6) | 0.87 | |
| Peripheral blast count, cells/μL | 0–5 | 1.0 | — | | |
| | >5–25 | 1.0 | (0.7–1.4) | 0.99 | 0.59 |
| | >25–100 | 1.21 | (0.9–1.7) | 0.23 | |
| | >100 | 1.31 | (0.7–2.5) | 0.41 | |
| Marrow blasts, % | 0–20 | 1.0 | — | | |
| | >20–50 | 0.96 | (0.7–1.4) | 0.81 | 0.76 |

© 2017 Sorror ML, et al. *JAMA Oncology.*

| | | | | | |
|---|---|---|---|---|---|
| | >50 | 1.06 | (0.8–1.5) | 0.73 | |

Covariates with different levels of severity and with an overall p-value are in blue.

GERD indicates gastro-esophageal reflux disease; ANC, absolute neutrophil count; LDH, lactate dehydrogenase; BMI, body mass index; WBC, and white blood count.

© 2017 Sorror ML, et al. *JAMA Oncology.*

**Supplemental eTable 8.** Components of the HCT-CI, Augmented HCT-CI, and the AML-CM and their Corresponding Scores

| Comorbidity | Definition | Score |
|---|---|---|
| **The HCT-CI** | | |
| Arrhythmia | Any type of arrhythmia that has necessitated the delivery of a specific anti-arrhythmia treatment at any time point in the patient's past medical history. | 1 |
| Cardiac | Coronary artery disease,[§] congestive heart failure, myocardial infarction, or EF ≤50% | 1 |
| Inflammatory bowel disease | Crohn's disease or ulcerative colitis requiring treatment at any time point in patient's past medical history. | 1 |
| Diabetes | Requiring treatment with insulin or oral hypoglycemic agents continuously for 4 weeks before start of treatment | 1 |
| Cerebrovascular disease | Transient ischemic attack or cerebrovascular accident | 1 |
| Psychiatric disturbance | Any disorder requiring continuous treatments for 4 weeks before start of treatment | 1 |
| Hepatic, mild | Chronic hepatitis, bilirubin > ULN to 1.5 × ULN, or AST/ALT> ULN to 2.5 × ULN; using the closest value before start of treatment. | 1 |
| Obesity | Patients with a body mass index >35 kg/m2 for patients older than 18 years or a BMI-for-age of ≥ 95th percentile for patients of ≤ 18 years of age | 1 |
| Infection | Requiring antimicrobial treatment starting from before treatment and continued beyond day 0 | 1 |
| Rheumatologic | Requiring specific treatment at any time point in the patient's past medical history | 2 |
| Peptic ulcer | Based on prior endoscopic or radiologic diagnosis | 2 |
| Moderate/severe renal | Creatinine clearance <45, if not available then serum creatinine > 2 mg/dL using the closest value before start of treatment, on dialysis, or prior renal transplantation | 2 |
| Moderate pulmonary | Corrected $D_LCO$ (via Dinakara equation) and/or FEV1 of 66%-80% or dyspnea on slight activity | 2 |
| Prior malignancy | Any prior malignancy to AML treated at any time point in the patient's past history, | 3 |

© 2017 Sorror ML, et al. *JAMA Oncology.*

| Comorbidity | Definition | Score |
|---|---|---|
| | excluding non-melanoma skin cancer and other myeloid malignancies. | |
| Heart valve disease | Of at least moderate severity, prosthetic valve, or symptomatic mitral valve prolapse as detected by echocardiogram | 3 |
| Severe pulmonary | Corrected $D_LCO$ (via Dinakara equation) and/or FEV1 ≤ 65% or dyspnea at rest or requiring oxygen | 3 |
| Moderate/severe hepatic | Liver cirrhosis, bilirubin > 1.5 × ULN, or AST/ALT > 2.5 × ULN; using the closest value before start of treatment. | 3 |
| **The Augmented HCT-CI = all the above + the following** | | |
| Comorbidity | **Definition** | **Score** |
| Hypoalbuminemia | Value of <3.5 g/dL; using the closest value before start of treatment. | 1 |
| Thrombocytopenia | Value of <20,000 cells/μL; using the closest value before start of treatment | 1 |
| Elevated LDH | Values of >200-1000 U/L; using the closest value before start of treatment. | 1 |
| Elevated LDH | Value of >1000 U/L; using the closest value before start of treatment. | 2 |
| **The AML-CM = all the above + the following** | | |
| Component | **Definition** | **Score** |
| Age | 50-59 years old | 1 |
| Age | ≥60 years old | 2 |
| ELN cytogenetic/molecular risk groups | Intermediate | 1 |
| ELN cytogenetic/molecular risk groups | Adverse | 2 |

Abbreviations: HCT-CI = hematopoietic cell transplantation-comorbidity index; EF = ejection fraction; ULN= upper limit of normal; BMI = body mass index; $D_LCO$= diffusion capacity of carbon monoxide; FEV1 = forced expiratory volume in one second; g/dL =

© 2017 Sorror ML, et al. *JAMA Oncology*.

gram/ deciliter; μL = microliter; LDH = lactate dehydrogenase; U/L = unit/liter; AML-CM = acute myeloid leukemia- composite model; and ELN = European leukemia network.

§One or more vessel-coronary artery stenosis requiring medical treatment, stent, or bypass graft.

© 2017 Sorror ML, et al. *JAMA Oncology*.

**Supplemental eTable 9: Comparisons of Kaplan Meier Survival Rates per Different Risk Factors and Models.**

| AML-CI | | | HCT-CI | | | Augmented HCT-CI | | | AML-CM | | |
|---|---|---|---|---|---|---|---|---|---|---|---|
| Score | Patients, % | OS, % | Score | Patients, % | OS, % | Score | Patients, % | OS, % | Score | Patients, % | OS, % |
| 0-1 | 30 | 69 | 0 | 17 | 70 | 0-2 | 32 | 73 | 1–4 | 27 | 84 |
| 2 | 32 | 55 | 1-2 | 37 | 74 | 3-4 | 30 | 65 | 5–6 | 24 | 65 |
| 3 | 18 | 61 | 3-4 | 26 | 50 | 5-6 | 20 | 57 | 7–9 | 31 | 52 |
| ≥4 | 20 | 40 | ≥5 | 19 | 30 | ≥7 | 18 | 20 | ≥10 | 18 | 21 |
| **Age (groups)** | | | **Cytogenetics (groups)** | | | **KPS (groups)** | | | | | |
| Group, years | Patients, % | OS, % | Group | Patients, % | OS, % | Group, % | Patients, % | OS, % | | | |
| 20-49 | 26 | 78 | Favor | 17 | 83 | 90-100 | 41 | 71 | | | |
| 50-59 | 24 | 66 | Intermediate | 36 | 66 | 75-80 | 35 | 59 | | | |
| 60+ | 50 | 41 | Adverse | 47 | 45 | ≤70 | 24 | 38 | | | |

AML-CI indicates acute myeloid leukemia-comorbidity index; HCT-CI, hematopoietic cell transplantation-comorbidity index; AML-CM, acute myeloid leukemia-composite model; and KPS, Karnofsky performance status.

© 2017 Sorror ML, et al. *JAMA Oncology.*

**Supplemental eTable 10: Power Calculation**

| Prevalence of risk factor or group | Minimal detectable risk increment[1] (risk ratio) |
|---|---|
| 10% | 0.17 (1.5) |
| 20% | 0.13 (1.35) |
| 30% | 0.11 (1.3) |
| 40% | 0.10 (1.25) |

[1] with 80% power at 2-sided 0.05 level of significance and overall risk of 40%

© 2017 Sorror ML, et al. *JAMA Oncology.*

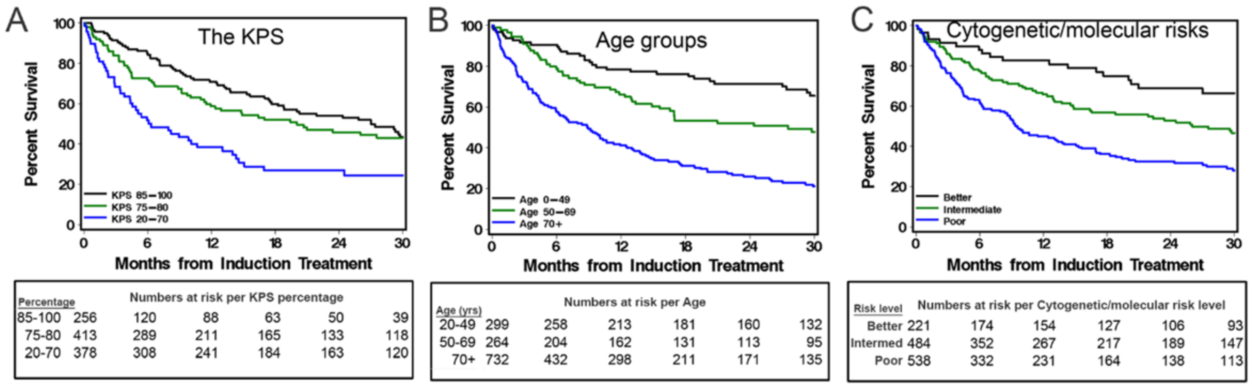


**eFigure 1: Kaplan Meier Estimates of Survival. Figure compares** Kaplan-Meier estimates of survival by **A)** Karnofsky performance status percentages, **B)** age groups, and **C)** Cytogenetic/molecular risk groups.

© 2017 Sorror ML, et al. *JAMA Oncology.*
